# Search for allosteric disulfide bonds in NMR structures

**DOI:** 10.1186/1472-6807-7-49

**Published:** 2007-07-20

**Authors:** Bryan Schmidt, Philip J Hogg

**Affiliations:** 1 UNSW Cancer Research Centre, University of New South Wales, Sydney, NSW 2052, Australia

## Abstract

**Background:**

Allosteric disulfide bonds regulate protein function when they break and/or form. They typically have a -RHStaple configuration, which is defined by the sign of the five chi angles that make up the disulfide bond.

**Results:**

All disulfides in NMR and X-ray protein structures as well as in refined structure datasets were compared and contrasted for configuration and strain energy.

**Conclusion:**

The mean dihedral strain energy of 55,005 NMR structure disulfides was twice that of 42,690 X-ray structure disulfides. Moreover, the energies of all twenty types of disulfide bond was higher in NMR structures than X-ray structures, where there was an exponential decrease in the mean strain energy as the incidence of the disulfide type increased. Evaluation of protein structures for which there are X-ray and NMR models shows that the same disulfide bond can exist in different configurations in different models. A disulfide bond configuration that is rare in X-ray structures is the -LHStaple. In NMR structures, this disulfide is characterised by a particularly high potential energy and very short α-carbon distance. The HIV envelope glycoprotein gp120, for example, is regulated by thiol/disulfide exchange and contains allosteric -RHStaple bonds that can exist in the -LHStaple configuration. It is an open question which form of the disulfide is the functional configuration.

## Background

It appears that introduction of disulfide bonds into proteins is an important mechanism by which they have evolved and are evolving [1-3]. A recent analysis of the trend in amino gain and loss in protein evolution showed that Cys have accrued in all 15 taxa studied [3]. In fact, Cys was the most frequently acquired amino acid in 8 of the 15 taxa. Considering that disulfide bonds will only form between optimally placed Cys in the tertiary structure, it follows that these bonds are a relatively recent addition to proteins.

Most protein disulfide bonds are motifs that stabilise the tertiary and quaternary protein structure. These bonds are also thought to assist protein folding by decreasing the entropy of the unfolded form [4]. A minor population of disulfide bonds serve a functional role. There are two types of functional disulfides; the catalytic and allosteric bonds.

The catalytic bonds are typically at the active sites of enzymes that mediate thiol/disulfide exchange in other proteins. These enzymes are the oxidoreductases [5,6]. The allosteric bonds, in contrast, control the function of the protein in which they reside by mediating a change when they break and/or form [7,8]. The type of change depends on the protein. It may be conformational as described for the HIV receptor, CD4 [9,10], or the resulting unpaired thiols of the cleaved allosteric bond may act as sites of alkylation by thiol modifiers as described for the blood clotting initiator, tissue factor [11,12]. The actions of the two functional disulfides are linked in that the redox state of the known allosteric disulfides are controlled by catalytic disulfides [9,12,13]. In an attempt to identify a common structural motif for allosteric disulfides the geometry and strain of 6,874 unique disulfide bonds in X-ray structures was recently examined [8].

A disulfide bond is made up of six atoms linking the two α-carbon atoms of the cysteine residues; C_α_-C_β_-S_γ_-S_γ_'-C_β_'-C_α_'. These six atoms define five chi angles, which are the rotation about the bonds linking the atoms. Each chi angle can be either positive or negative, which equates to 20 possible disulfide bond configurations. The three basic types of disulfide are the spirals, hooks or staples and depending on the sign of the χ_3 _angle they are either right- or left-handed [14]. We expanded these standard definitions to reflect the sign of the χ_1 _and χ_1_' torsional angles [8]. For instance, a disulfide is a minus right handed spiral (-RHSpiral) if the χ_1 _χ_2 _χ_3 _χ_2_' and χ_1_' angles are -, +, +, + and -, respectively. The disulfides are treated as symmetrical. For example, a disulfide is a +/-RHSpiral if the χ_1_, χ_2_, χ_3_, χ_2_', χ_1_' angles are +, +, +, +, - or -, +, +, +, +.

The spirals are the main structural disulfides. With one or two exceptions all the catalytic disulfides are +/-RHHooks, while the known allosteric disulfides are -RHStaples [8]. The allosteric bonds are also defined by closely-spaced α-carbon atoms of the two cysteine residues. The -RHStaple bonds have a mean α-carbon atom distance of 4.3 Å, compared to a mean of 5.6 Å for all disulfides [8]. This is because of their position in protein structures. These bonds often link adjacent strands in the same β-sheet secondary structure [7,15]. The strands are usually so close in the β-sheet that they need to pucker to accommodate the disulfide bond [15].

While most protein structures have been solved by X-ray crystallography, a growing number of NMR structures are becoming available. There are also some proteins whose structure has been determined by both methods. A recent analysis of 78 protein structures determined by both X-ray and NMR methods showed that 18 of the 78 structures are significantly different, while the other 60 structures are very similar [16]. The large scale differences likely reflect crystal versus solution structures.

The primary limitation in determining protein structure by NMR is the size of the protein. The size limitation for complete atomic-resolution structure determination by NMR is currently ~30 kDa, though backbone assignments and general folds have been described for proteins up to 100 kDa. X-ray crystallography does not suffer from the size restrictions of NMR, with protein size having no direct bearing on the solvability of the protein or protein complex. This is at least partly why most protein structures have been determined by X-ray rather than NMR. The limitation of X-ray crystallography is its static nature. This means that only a single structure can be determined and any protein movement during data collection results in decreased resolution. Indeed, in many structures there are segments of the protein that are so disordered they are not contained in the structure. With the advent of time-resolved crystallography some dynamic data can be obtained. However, each individual snapshot is still limited by the requirement of an unmoving structure.

In this study, we compare and contrast the disulfide configurations and energies of all NMR and X-ray protein structures. Analysis of the points of contrast between the datasets have led to the identification of a new potential allosteric disulfide defined by the -LHStaple configuration.

## Results and discussion

As of June 20, 2006, there were 37,141 structure files available in the protein databank. Of these, 31,611 were determined by X-ray crystallography, 5,476 were determined by NMR and 54 were determined by cryo-electron microscopy or powder diffraction. There was a mean of 15 structural models in each NMR file deposited, resulting in 84,584 total NMR structural models. There were 97,741 disulfides in all files, as determined by the presence of an SSBOND line in the PDB file. Of these disulfides, 42,690 were found in X-ray structures, 55,005 in the separate NMR structures, and 46 were from structures determined by the other methods.

There is a mean of 1.4 disulfide bonds listed per X-ray structure file in the PDB. This is higher than the mean of 0.6 disulfide bonds per NMR structure and 0.9 disulfide bonds per structure determined by other methods. The prototypical structural disulfide configuration, the -LHSpiral [8], accounts for nearly 30% of all disulfides in X-ray structures (Table 1) and 20% of the disulfides in NMR structures (Table 2).

**Table 1:** Distribution and strain energies of 42,690 disulfides in X-ray structures.

**Designation**	**Number**	**Incidence, %**	**DSE, kJ.mol**^-1^	**Cα-Cα', Å**
-LHSpiral	12684	29.71	10.5 (10.4–10.6)	5.77 (5.76–5.78)
-RHHook	4344	10.18	13.3 (13.1–13.6)	5.26 (5.22–5.30)
-RHStaple	3641	8.53	17.7 (17.5–17.9)	4.18 (4.17–4.19)
+/-LHSpiral	3563	8.35	13.2 (12.9–13.4)	6.11 (6.09–6.12)
-/+RHHook	2445	5.73	11.3 (11.0–11.5)	5.09 (5.07–5.10)
+/-RHSpiral	2392	5.60	13.6 (13.3–13.9)	6.16 (6.06–6.26)
-RHSpiral	2311	5.41	11.9 (11.6–12.3)	5.75 (5.71–5.78)
-LHHook	2262	5.30	14.6 (14.2–15.0)	5.65 (5.62–5.68)
+/-RHHook	2051	4.80	14.1 (13.7–14.5)	5.39 (5.28–5.49)
-/+LHHook	1949	4.57	12.9 (12.5–13.3)	5.96 (5.92–5.99)
+RHSpiral	1599	3.75	15.7 (15.4–15.9)	6.43 (6.41–6.44)
+/-LHHook	763	1.79	17.1 (16.3–17.9)	5.47 (5.42–5.52)
+/-LHStaple	618	1.45	15.3 (14.3–16.2)	5.18 (5.09–5.27)
-LHStaple	599	1.40	14.9 (13.8–16.0)	5.80 (5.70–5.89)
+LHHook	451	1.06	17.2 (16.2–18.2)	5.87 (5.81–5.94)
+/-RHStaple	301	0.71	19.0 (17.9–20.2)	5.11 (5.03–5.19)
+LHSpiral	293	0.69	18.2 (16.9–19.5)	6.35 (6.30–6.40)
+RHHook	269	0.63	20.8 (19.4–22.2)	5.91 (5.83–5.99)
+LHStaple	109	0.26	12.2 (9.7–14.6)	5.66 (5.54–5.77)
+RHStaple	46	0.11	33.0 (29.3–36.7)	5.94 (5.63–6.26)
all disulfides	42690		13.1 (13.1–13.2)	5.59 (5.58–5.60)

The disulfide bonds were separated into twenty configurations based on the sign of χ_1, _χ_2_, χ_3_, χ_2_' and χ_1_' angles [8]. The dihedral strain energy (DSE) and distance between the two α carbon atoms were calculated for each disulfide and the mean and 95% confidence intervals is shown for each group.

**Table 2:** Distribution and strain energies of 55,005 disulfides in NMR structures.

**Designation**	**Number**	**Incidence, %**	**DSE, kJ.mol**^-1^	**Cα-Cα', Å**
-LHSpiral	11137	20.25	19.2 (19.0–19.5)	5.73 (5.72–5.74)
-RHHook	7087	12.88	31.2 (30.8–31.6)	5.80 (5.79–5.82)
-LHHook	5313	9.66	34.5 (34.0–34.9)	5.50 (5.47–5.52)
+/-RHSpiral	4106	7.46	21.9 (21.5–22.3)	5.85 (5.83–5.87)
-RHSpiral	3689	6.71	27.2 (26.7–27.7)	6.16 (6.14–6.18)
-RHStaple	3150	5.73	26.0 (25.5–26.4)	4.49 (4.46–4.52)
+/-LHSpiral	3025	5.50	24.2 (23.7–24.6)	6.06 (6.04–6.08)
-/+RHHook	2527	4.59	23.3 (22.7–23.9)	5.42 (5.38–5.54)
+/-RHHook	2318	4.21	27.3 (26.7–27.9)	5.65 (5.62–5.68)
+RHSpiral	2064	3.75	20.2 (19.6–20.7)	5.79 (5.77–5.81)
+/-LHHook	2057	3.74	29.6 (29.0–30.3)	5.81 (5.78–5.84)
-/+LHHook	2001	3.64	31.0 (30.4–31.6)	5.79 (5.75–5.82)
+/-LHStaple	1899	3.45	29.6 (29.0–30.1)	5.06 (5.02–5.10)
-LHStaple	1805	3.28	36.1 (35.4–36.7)	4.88 (4.84–4.93)
+/-RHStaple	889	1.62	29.2 (28.3–30.0)	5.28 (5.23–5.33)
+LHHook	606	1.10	29.3 (28.2–30.3)	5.89 (5.84–5.94)
+RHHook	530	0.96	31.3 (29.8–32.3)	5.92 (5.86–5.97)
+LHSpiral	342	0.62	29.2 (27.6–30.8)	6.20 (6.15–6.25)
+LHStaple	256	0.47	27.9 (25.9–29.8)	5.38 (5.26–5.49)
+RHStaple	204	0.37	34.0 (32.4–35.6)	5.19 (5.08–5.31)
all disulfides	55005		26.5 (26.3–26.6)	5.64 (5.63–5.64)

The disulfide bonds were separated into twenty configurations based on the sign of χ_1, _χ_2_, χ_3_, χ_2_' and χ_1_' angles [8]. The dihedral strain energy (DSE) and distance between the two α carbon atoms were calculated for each disulfide and the mean and 95% confidence intervals is shown for each group.

**Figure 1:**
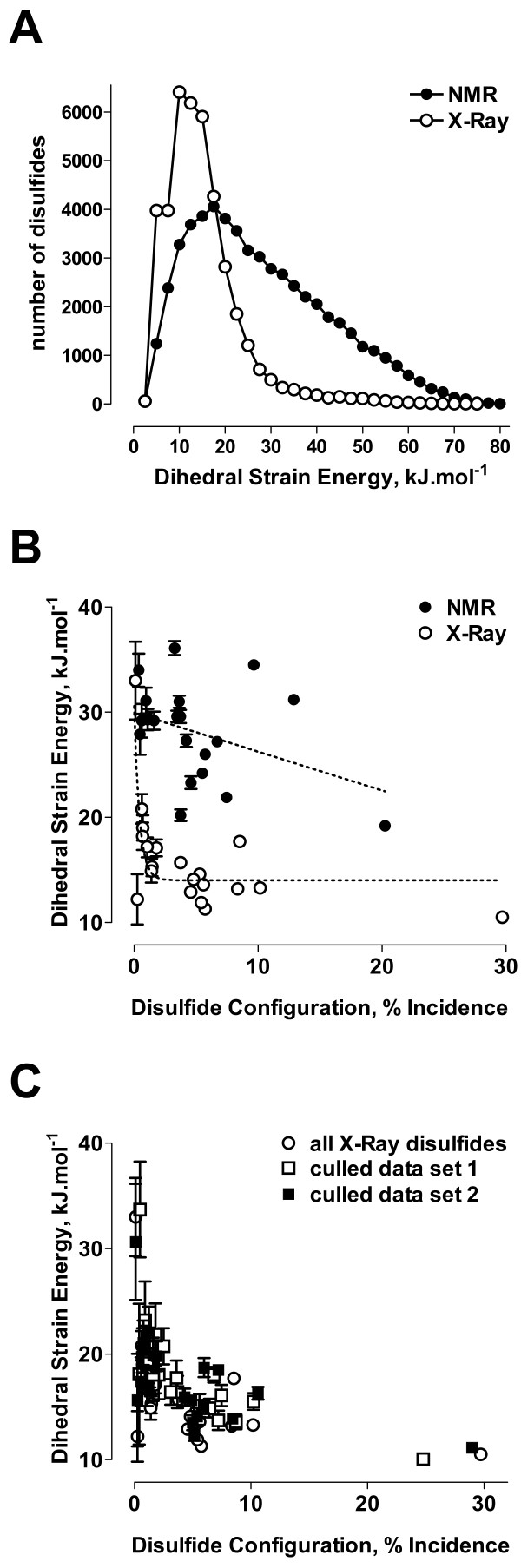
**Distribution of disulfide strain energies in NMR and X-ray structures**. A. Number of disulfide bonds for each dihedral strain energy (in 2.5 kJ.mol^-1 ^increments) for structures determined by NMR (total of 55,005 disulfides, Table 2) and X-ray (total of 42,690 disulfides, Table 1). B. Plot of the mean strain energy and 95% confidence intervals of each disulfide configuration versus the incidence of that configuration. The dotted lines are the linear least-squares fit to the NMR data (top line; Table 2) or single exponential least squares fit to the X-ray data (bottom line; Table 1). C. Plot of the mean strain energy and 95% confidence intervals of each disulfide configuration versus the incidence of that configuration for all X-ray disulfides (42,690 disulfides; see part B), a unique set of 6,874 X-ray disulfides described by Schmidt et al. [8] (data set 1) and the 16,225 disulfides of a culled set of X-ray structures described by Guoli Wang and Roland Dunbrack, Jr. [25] (data set 2).

The five chi angles of the disulfide bond was used to estimate the potential energy of each bond, or dihedral strain energy [8,17,18]. This energy measurement is approximate but has been shown to be a useful measure of disulfide strain [19-22]. A striking feature is the disparity in dihedral strain energy between NMR and X-ray disulfides. The mean dihedral strain energy of all NMR disulfides (26.5 kJ.mol^-1^, Table 2) is twice that of X-ray disulfides (13.1 kJ.mol^-1^, Table 1). The ordering of the mean strain energies between the different dihedral configurations, though, is nearly the same between NMR and X-ray structures. This supports the validity of the analysis and highlights the difference in tolerance for highly strained disulfides in NMR versus X-ray structures. This is demonstrated graphically in Fig. 1A, where the dihedral strain energies of disulfides in NMR structures have a much broader distribution across the energy range. In NMR structures there is only a modest linear decrease in the mean strain energy as a function of the incidence of each disulfide configuration. In X-ray structures, however, there is an exponential decrease in the mean strain energy as the incidence of the configuration increases (Fig. 1B). The overall spread of values is similar, however, with the strain energies ranging from 2.1 to 79.1 kJ.mol^-1 ^in NMR structures and from 2.1 to 75.6 kJ.mol^-1 ^in X-ray structures.

There are several possible explanations for the higher average strain energy of disulfide bonds in NMR-determined structures. One possibility is a higher degree of error in defining disulfide bond structures in NMR compared to X-ray structures. To test this notion, the disulfide bonds in a dataset of uniformly refined NMR structures [23,24] was analysed.

Of the 100 validated structures, 25 contain one or more disulfide bonds (PDB IDs 1b2t, 1bbn, 1bf0, 1bf9, 1bgk, 1ce3, 1chl, 1cw5, 1cw6, 1df6, 1du9, 1e5b, 1e5c, 1e8p, 1e8q, 1efe, 1eig, 1eih, 1eot, 1eph, 1epj, 1eww, 1fgp, 1fo7 and 1fwo). There is a total of 60 disulfides in the 25 summary structures and 713 total disulfides in all individual models. As for the total NMR structures dataset (Table 2), the -LHSpiral is the most common disulfide in these refined structures, representing 15 of the 60 disulfides in the summary structures and 185 of the 713 disulfides in all individual models. Notably, the mean dihedral strain energy of the -LHSpiral disulfides in the refined structures (n = 713; 21.3 kJ.mol^-1^; 95% CI, 19.8–22.9 kJ.mol^-1^) is almost the same as it is for all NMR structures (n = 11137, 19.2 kJ.mol^-1^; 95% CI, 19.0–19.5 kJ.mol^-1^, Table 2). This strain energy is roughly twice that found for -LHSpiral disulfides in X-ray structures (n = 12684, 10.5 kJ.mol^-1^; 95% CI, 10.4–10.6 kJ.mol^-1^, Table 1) [8]. Thus, while it is likely that there are errors in the modelling of both NMR and X-ray structures, particularly for disulfides with high strain, the significant differences noted in average strain energies of disulfides in NMR versus X-ray structures most probably indicate preference for lower energy disulfides in crystallized proteins.

The lower tolerance for disulfide strain energy in X-ray structures is also apparent when comparing the data for all X-ray structures in Table 1 with the data we reported earlier for a set of unique X-ray disulfides [8] and the disulfides of a culled set of X-ray structures described by Guoli Wang and Roland Dunbrack, Jr. [25] (Table 3, Fig. 1C). The Wang and Dunbrack structures represent non-redundant sequences across all PDB files and were selected based on the highest resolution structure available and then the best R-values. The overall trend in relative strain energies of the different configurations and their incidence is the same for the non-culled and culled datasets. This finding indicates that the analysis of the non-culled dataset has not been unduly biased by those proteins for which there are numerous X-ray structures, such as serine proteinases like trypsin.

**Table 3:** Distribution and strain energies of 16,225 disulfides of a culled set of X-ray structures described by G. Wang and R. Dunbrack, Jr. (file pdbaanr) [25].

**Designation**	**Number**	**Incidence, %**	**DSE, kJ.mol**^-1^	**Cα-Cα', Å**
-LHSpiral	4697	28.95	11.1 (10.9–11.4)	5.74 (5.73–5.75)
-RHHook	1718	10.59	16.3 (15.8–16.9)	5.36 (5.30–5.41)
+/-LHSpiral	1366	8.42	13.9 (13.5–14.3)	6.14 (6.12–6.16)
-RHStaple	1168	7.20	18.5 (18.1–18.9)	4.22 (4.19–4.25)
-LHHook	969	5.97	18.7 (17.9–19.6)	5.58 (5.53–5.63)
+/-RHSpiral	961	5.92	15.0 (14.4–15.6)	6.05 (6.02–6.08)
-RHSpiral	873	5.38	14.1 (13.3–14.9)	5.82 (5.79–5.85)
-/+RHHook	834	5.14	12.4 (11.8–13.0)	5.11 (5.08–5.15)
-/+LHHook	811	5.00	13.5 (12.8–14.2)	6.01 (5.96–6.06)
+RHSpiral	794	4.89	15.6 (15.2–16.1)	6.36 (6.34–6.39)
+/-RHHook	698	4.30	15.9 (15.2–16.7)	5.41 (5.24–5.58)
+/-LHHook	309	1.90	19.8 (18.4–21.3)	5.57 (5.48–5.66)
+/-LHStaple	261	1.61	19.9 (18.2–21.5)	5.20 (5.07–5.32)
-LHStaple	202	1.24	22.1 (19.7–24.5)	5.39 (5.25–5.54)
+LHHook	173	1.07	16.8 (15.1–18.5)	5.91 (5.82–6.00)
+/-RHStaple	125	0.77	21.1 (19.0–23.2)	5.21 (5.09–5.33)
+RHHook	116	0.71	20.4 (18.3–22.5)	5.83 (5.72–5.94)
+LHSpiral	92	0.57	17.4 (15.0–19.7)	6.33 (6.26–6.41)
+LHStaple	42	0.26	15.7 (11.2–20.1)	5.64 (5.37–5.92)
+RHStaple	16	0.10	30.6 (25.1–36.2)	5.31 (4.77–5.86)
all disulfides	16225		14.6 (14.5–14.8)	5.62 (5.60–5.63)

The disulfide bonds were separated into twenty configurations based on the sign of χ_1, _χ_2_, χ_3_, χ_2_' and χ_1_' angles [8]. The dihedral strain energy (DSE) and distance between the two α carbon atoms were calculated for each disulfide and the mean and 95% confidence intervals is shown for each group.

Direct comparison of disulfide bond characteristics in NMR and X-ray structures can be made for proteins whose structures have been determined by both methods. The disulfide bond configurations in 10 proteins that have very similar X-ray and NMR structures (MaxSub ≥ 0.77) has been determined (Table 4). The differences in the X-ray versus NMR models of the proteins is comparable to the differences between various X-ray or various NMR structures of a given protein [16]. It is apparent that a given disulfide can exist in different configurations in NMR models. Most often, the configuration found in the X-ray structure is also found in one or more of the NMR models. For example, the Cys26–Cys84 disulfide in ribonuclease A is a -LHSpiral in the X-ray structure and in 16 of the 32 NMR models. In the other 16 models it is a -RHHook (13) or -RHSpiral (3). There are some notable exceptions however. The Cys11–Cys27 disulfide in tendamistat is a -/+RHHook in the X-ray structure and a +/-LHStaple in all 9 NMR models. Also, the Cys25–Cys80 disulfide in β_2_-microglobulin is a -LHStaple in the X-ray structure but a -LHSpiral (10), -RHSpiral (7) or -RHHook (3) in the 20 NMR models. These findings indicate that structures of some disulfides are particularly malleable.

**Table 4:** Comparison of the disulfide bond configurations in proteins that have very similar X-ray and NMR structures.

**Protein**	**X-Ray**					**NMR**					**Structural Similarity**	
	PDB	resolution	disulfides	configuration	DSE, kJ.mol^-1^	PDB	models	disulfides	configuration^1^	DSE, kJ.mol^-1^	RMSD^3^	MaxSub^4^
thioredoxin	2tir	2	32–35	+/-RHHook		1xoa	20	32–35	-RHHook (15)		0.93	0.93
									+/-RHHook (5)			
ferredoxin II	1fxd	1.7	18–42	+/-RHSpiral		1f2g	15	18–42	-RHHook (5)		0.96	0.93
									+/-LHStaple (5)			
									+/-RHSpiral (5)			
ribonulcease A	1kf5	1.1	26–84	-LHSpiral	7.1	2aas	32	26–84	-LHSpiral (16)	12.6 (11.8–13.3)^2^	1.1	0.92
									-RHHook (13)			
									-RHSpiral (3)			
			40–95	-LHSpiral	4.3			40–95	-LHSpiral (32)	4.1 (3.4–4.8)		
			58–110	-LHSpiral	10.7			58–110	-LHSpiral (30)	11.4 (11.1–11.7)		
									-RHHook (2)			
			65–72	-LHHook				65–72	-RHHook (32)			
ovomucoid	2ovo	1.5	8–38	-LHSpiral	6.1	1tur	12	8–38	-LHSpiral (12)	23.0 (20.6–25.4)	2.04	0.89
			16–35	+/-LHSpiral				16–35	-LHSpiral (11)			
									-RHSpiral (1)			
			24–56	-RHHook	6.4			24–56	-RHHook (6)	11.1 (10.4–11.8)		
									-LHHook (3)			
									-LHStaple (3)			
tendamistat	1hoe	2	11–27	-/+RHHook		2ait	9	11–27	+/-LHStaple (9)		3.57	0.87
			45–73	-LHSpiral				45–73	-RHHook (3)			
									-RHSpiral (2)			
									-LHSpiral (1)			
									+/-LHStaple (1)			
									+/-RHHook (1)			
									-LHHook (1)			
erabutoxin B	3ebx	1.4	3–24	-LHSpiral	2.8	1fra	14	3–24	-LHSpiral (11)	23.2 (13.7–32.7)	1.42	0.87
									-LHHook (2)			
									-RHHook (1)			
			17–41	-LHSpiral				17–41	-RHHook (11)			
									-LHHook (1)			
									-/+LHHook (1)			
									+/-RHHook			
			43–54	-RHSpiral				43–54	-LHSpiral (6)			
									-RHSpiral (5)			
									-RHHook (2)			
									-RHStaple (1)			
			55–60	+RHSpiral	10.4			55–60	+RHSpiral (9)	38.1 (35.5–40.6)		
									+/-RHSpiral (4)			
									+LHSpiral (1)			
lipid transfer protein	1mzl	1.9	4–52	-LHSpiral		1afh	15	4–52	+/-RHSpiral (10)		1.67	0.84
									-RHHook (3)			
									-RHSpiral (2)			
			14–29	+/-RHHook				14–29	+/-LHStaple (5)			
									+/-RHHook (3)			
									-LHStaple (3)			
									-/+LHHook (2)			
									-LHHook (2)			
			30–75	-LHSpiral	4.4			30–75	-LHSpiral (12)	23.9 (22.4–25.3)		
									-LHHook (2)			
									-RHHook (1)			
			50–89	+/-LHSpiral				50–89	+/-LHHook (6)			
									-LHSpiral (5)			
									+/-LHSpiral (3)			
									+/-RHHook (1)			
β-lactoglobulin	1bsy	2.2	66–160	-RHSpiral	9.3	1dv9	21	66–160	-RHSpiral (4)	39.9 (29.5–50.3)	2.36	0.83
									-LHSpiral (3)			
									+/-LHSpiral (3)			
									-/+LHHook (3)			
									+/-RHStaple (2)			
									-LHHook (2)			
									+/-RHSpiral (1)			
									+/-RHHook (1)			
									+/-LHStaple (1)			
									-/+RHHook (1)			
			106–119	-RHStaple	15.8			106–119	-RHStaple (12)	18.6 (17.1–20.2)		
									-LHStaple (8)			
									-LHHook (1)			
ribonulcease T1	4rnt	2.2	2–10	-LHHook	15.3	1ygw	34	2–10	-LHHook (16)	26.6 (26.5–26.8)	1.82	0.82
									+/-RHStaple (10)			
									-/+RHHook (4)			
									-RHStaple (3)			
									-RHHook (1)			
			6–103	-RHStaple	11.7			6–103	-RHStaple (28)	28.9 (25.8–32.1)		
									-RHHook (6)			
β_2_-microglobulin	1lds	1.8	25–80	-LHStaple		1jnj	20	25–80	-LHSpiral (10)		3.46	0.77
									-RHSpiral (7)			
									-RHHook (3)			

1 Numbers in brackets are the number of disulfides with that configuration.

2 The mean dihedral strain energy (DSE) and 95% confidence intervals.

3 The root-mean square deviations (RMSD) value was calculated between all C_α _atoms of the X-ray structure and the first NMR model [16].

4 MaxSub is a measure of structural similarity of the X-ray and NMR structures [16]. A score of 1.0 means that all C_α _atoms are matched between the X-ray and NMR structures, while a score towards zero indicates very different structures. All the structures listed in the table have only small-scale differences (MaxSub values from 0.77 to 0.93).

There are 10 disulfides in this dataset of comparable structures where the X-ray configuration is also the predominant NMR configuration. Notably, nine of the ten dihedral strain energies for the matching disulfide configurations are significantly higher in NMR structures (Table 4). This finding supports the notion that the propensity for a protein to crystallize relates, at least in part, to the amount of strain in its disulfide bonds.

**Figure 2:**
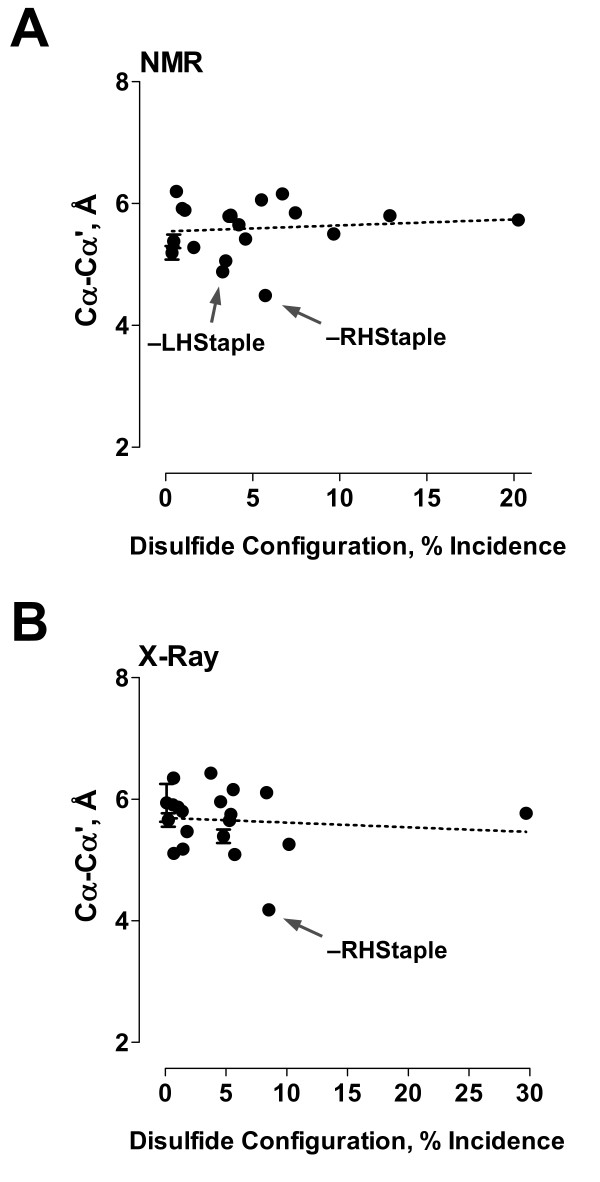
**Mean distance between the α-carbons of each of the 20 disulfide configurations in NMR and X-ray structures**. The mean distance between the α carbons of all disulfides is 5.6 Å for both NMR (part A) and X-ray (part B) structures. The outliers with a short α carbon distance are the allosteric -RHStaple bonds in both NMR and X-ray structures and the -LHStaple bonds in NMR structures. The dotted lines are the linear least-squares fit to the data.

The mean distance between the α-carbon atoms of the disulfide bond is the same in NMR and X-ray structures, at 5.6 Å (Tables 1 and 2). The -RHStaple configuration is the standout for α-carbon distance, with mean distances of 4.5 Å and 4.2 Å in NMR and X-ray structures, respectively (Fig. 2). As discussed previously [8,15], this is because -RHStaples are often found linking adjacent strands in the same antiparallel β-sheet. The -RHStaple configuration is favoured by allosteric disulfides [8]. The finding that -RHStaples have the same features in NMR and X-ray structures further supports this motif as a hallmark of allosteric bonds. The catalytic disulfides in X-ray structures are nearly always +/-RHHooks [8]. They are also predominantly +/-RHHooks in NMR structures of oxidoreductases (data not shown), but can exist in subsets of the RHHook configuration. The catalytic disulfide in one NMR structure of thioredoxin (PDB ID 1xoa), for example, is a -RHHook in 15 of the 20 models and a +/-RHHook in the other 5 (Table 4).

**Figure 3:**
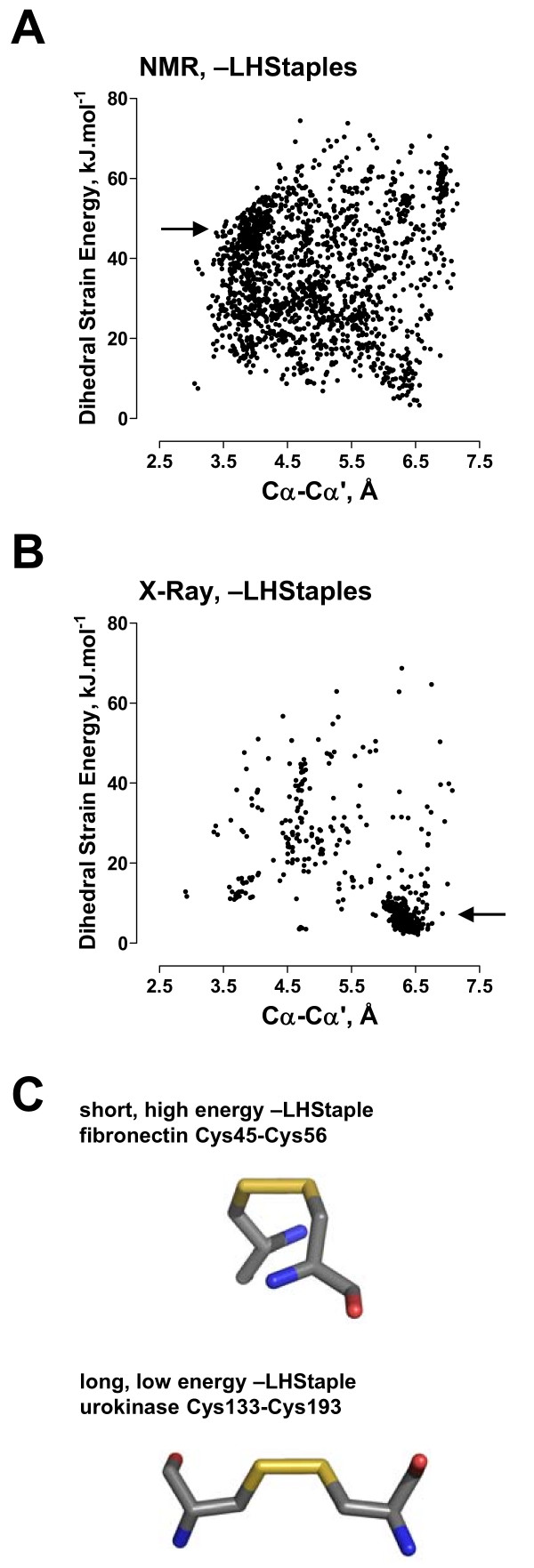
**Distribution of strain energies and α-carbon distances for the -LHStaple disulfides in NMR and X-ray structures**. A major fraction of the 1,805 -LHStaple bonds in NMR structures (part A) have a high strain energy (~50 kJ.mol^-1^) and short α-carbon distance (~4 Å). The majority of the 599 -LHStaple bonds in X-ray structures (part B) have a low strain energy (~10 kJ.mol^-1^) and long α-carbon distance (~6.5 Å). Example of a short, high energy -LHStaple (the Cys45–Cys56 bond in fibronectin, PDB ID 1o9a) and a long, low energy -LHStaple (the Cys133–Cys193 bond in urokinase plasminogen activator, PDB ID 2fd6) is shown in part C. The fibronectin disulfide is a NMR structure (Table 4), while the urokinase plasminogen activator disulfide is a X-ray structure with a resolution of 1.9 Å, a DSE of 2.9 kJ.mol^-1 ^and an α-carbon distance of 6.5 Å. The structures look at the side of the S-S bond, which is shown in the horizontal position. They were generated using PyMol [35].

While the average features of most configurations are generally comparable between NMR and X-ray structures, the features of the -LHStaple bond are very different between the two. Overall, the -LHStaples in NMR structures have a mean strain energy of 36.1 kJ.mol^-1 ^(n = 1805; 95% CI, 35.4–36.7 kJ.mol^-1^) and a mean Cα-Cα' distance of 4.88 Å (95% CI, 4.84–4.93 Å). This is compared to a mean strain energy of 14.9 kJ.mol^-1 ^(n = 599; 95% CI, 13.8–16.0 kJ.mol^-1^) and a mean Cα-Cα' distance of 5.80 Å (95% CI, 5.70–5.89 Å) for this configuration in X-ray structures. From visual inspection of all the -LHStaples (Fig. 3), it is apparent that the majority of these bonds in NMR structures have a high strain energy (~50 kJ.mol^-1^) and short α-carbon distance (~4 Å) (Fig. 3A). In contrast, most of these bonds in X-ray structures have a low strain energy (~10 kJ.mol^-1^) and long α-carbon distance (~6.5 Å) [8] (Fig. 3B).

Due to the high strain energies of these short -LHStaples, it is understandable that they would be rare in X-ray structures due to the generally low tolerance for high energy bonds. In NMR and X-ray structures that contain -RHStaple disulfides, it is apparent that these bonds can often exist in the -LHStaple configuration and vice versa. Moreover, the disulfides that can exist in both -RHStaple and -LHStaple configurations almost invariably have high strain energy and a short α-carbon separation in both the right-handed and left-handed configurations (data not shown). These findings suggest that the -LHStaple should be considered a potential allosteric bond. Indeed, it remains in question if it is the -RHStaple or the strained -LHStaple that is the functional form of allosteric disulfide bonds. Two proteins in which this switching occurs, fibronectin and HIV gp120, will be discussed in more detail.

Fibronectin is a major component of extracellular matrices where it influences a variety of cellular functions by binding to surface integrin receptors [26]. Following secretion from cells it assembles into a fibrillar network that once formed is resistant to all denaturants except reducing agents [27]. The mechanism of fibril formation is not well understood but it may involve domain swapping [28,29]. The five N-terminal type 1 repeats of fibronectin are essential for fibril formation [26]. Type 1 domains are ~40 residues in length and contain two disulfide bonds in a 1–3, 2–4 pattern. The 1–3 disulfide in each domain can exist in hook or spiral configurations, while the 2–4 disulfide is always a -RHStaple or -LHStaple with a very short α-carbon distance of ≤ 4 Å (Table 5). Given the apparent necessity for a -RHStaple or -LHStaple in the 2–4 disulfides, we suggest that these are allosteric disulfides that might regulate fibril formation. The fact that the -LHStaple configuration of these bonds uniformly have a higher DSE and shorter α-carbon separation than the -RHStaple configuration can be interpreted to suggest either that there is some uniform defect in the modelling of this configuration or that the -LHStaple is the functional configuration.

**Table 5:** Possible allosteric disulfides in human fibronectin.

**Domain**	**Number**	**Disulfide**	**Configuration**	**DSE, kJ.mol**^-1^	**Cα-Cα', Å**	**PDB ID**
Type 1	1st	21–47^1^	-/+RHHook	55.7	6.14	1o9a
			-RHSpiral	24.8	6.32	1qgb
		45–56	-LHStaple	46.2	3.91	1o9a
			-RHStaple	21.5	4.03	1qgb
Type 1	2nd	66–94	-/+RHHook	71.2	6.70	1o9a
			+LHHook	40.5	6.51	1qgb
		92–104	-RHStaple	32.2	4.00	1o9a
			-LHStaple	48.4	3.88	1qgb
Type 1	4th	155–184	+/-RHSpiral	27.9	5.59	1fbr
		182–194	-RHStaple	21.6	3.78	1fbr
Type 1	5th	200–229	-RHSpiral	12.4	5.59	1fbr
		227–239	-RHStaple	23.3	3.83	1fbr
Type 1	6th	277–304	+/-RHSpiral	25.6	6.09	1qo6
		302–311	-RHStaple	23.2	3.60	1qo6
Type II	1st	329–355	+/-LHHook	12.2	5.85	1qo6
		343–370	-LHSpiral	25.1	6.23	1qo6

Four summary NMR structures from PDB IDs 1o9a, 1qgb, 1fbr and 1qo6 were used for this analysis.

1 Numbering for mature protein (2,446 residues).

The HIV envelope glycoprotein consists of the surface glycoprotein gp120 bound non-covalently to transmembrane gp41 that is anchored in the viral membrane [30]. The two proteins dissociate when gp120 binds to CD4 and a chemokine receptor. This allows the gp41 fusion peptide to be inserted into the target membrane, which drives the membrane merger [31]. Cleavage of two of the nine disulfide bonds in gp120 appears to be important in this process [32,33]. It has been proposed that cleavage of the gp120 bonds facilitate unmasking of the gp41 fusion peptide and its insertion into the target cell membrane [32,33]. Seven of the nine disulfide bonds are present in the eight core structures of gp120 in the protein databank, and five of these bonds can exist in either -RHStaple or -LHStaple configurations in the different structures (Table 6). Considering that the V3 domain binds chemokine receptor and that cleavage of gp120 disulfides ablates this interaction [32], the Cys296–Cys331 bond that tethers the ends of V3 is most likely one of the two disulfides cleaved in gp120. There is currently no experimental data to suggest what other disulfide is cleaved. Our analysis leads us to propose that the Cys385–Cys418 disulfide is the other bond cleaved.

**Table 6:** Features of the HIV gp 120 disulfide bonds

**Disulfide**^1^	**Domain**	**Configurations**^2^	**Overall DSE, kJ.mol**^-1^	**Overall Cα-Cα', Å**
119–205	spans V1/V2	-LHHook (3) +/-RHHook (2) -LHStaple (1) -RHSpiral (1) -/+LHHook (1)	23.5 ± 4.7^3^	5.02 ± 0.13^4^
126–196	spans V1/V2	-RHStaple (7) -RHHook (1)	25.2 ± 2.4	4.37 ± 0.11
218–247	within C2	-RHStaple (8)	15.4 ± 1.0	3.84 ± 0.04
228–239	within C2	-RHSpiral (5) +RHSpiral (2) +/-RHSpiral (1)	12.2 ± 0.4	6.02 ± 0.06
296–331	spans V3	-RHStaple (8)	14.6 ± 1.0	3.86 ± 0.04
378–445	spans V4	+/-RHSpiral (4) -RHSpiral (3) -/+RHHook (1)	17.3 ± 2.2	6.30 ± 0.08
385–418	spans V4	-LHHook (5) -RHStaple (2) -LHStaple (1)	28.2 ± 3.7	3.79 ± 0.03

1 Nunbering for the gp 160 precursor protein.

2 Eght X-ray structures from PDB IDs 1g9m, 1g9n, 1gcn, 1rzj, 1rzk, 1yy1, 1yym and 2b4c were used for this analysis from. Numbers in brackets are the number of disulfides with that configuration.

3 The error is ± SE.

The Cys126–Cys196 bond is found in the -RHStaple configuration in seven of the eight structures and has strain energies ranging from 20 to 40 kJ.mol^-1 ^(Table 5). However, the distance between α-carbons for this bond is longer than for the other -RHStaples in this protein. The Cys218–Cys247 is also found in the -RHStaple configuration in the solved structures and the α-carbon separation is less than 4 Å. The strain energies for this bond are modest, though, ranging from 12 to 20 kJ.mol^-1^. By comparison, the Cys385–Cys418 bond is found as a -RHStaple in two of the reported structures and as a -LHStaple in one structure. In the remaining structures, it is found as a -LHHook. The strain energies are around 30 kJ.mol^-1^, however, with the -LHStaple configuration having a strain of 43 kJ.mol^-1^. Additionally, the α-carbon separation is short, ranging from 3.7 to 3.9 Å in all of the structures. While the predominant configuration of this bond, -LHHook, has not been associated with allosteric disulfides, the high strain of this bond disposes it to cleavage. Although, given the preference for lower energy bond configurations in X-ray structures, it is possible that the predominance of the -LHHook configuration in this structure is a biproduct of crystal packing. We suggest that it is the -LHStaple configuration of this bond that is most susceptible to cleavage and is the second disulfide cleaved during viral entry. The Cys385–Cys418 bond is in the same β-barrel as the Cys296–Cys331 disulfide. It is plausible that accessibility of one of these bonds to the reductant leads to the accessibility of the other bond as well. The cleavage of two strained, cross-strand disulfides in one structural motif should allow for a large conformational change in the domain.

## Conclusion

Comparison of the same disulfide bonds in very similar X-ray and NMR structures indicates that the bonds often exist in different configurations in different NMR models and usually with a higher potential energy than found in X-ray structures. One bond configuration that is scarce in X-ray structures is the -LHStaple. In NMR structures, this disulfide is characterised by a particularly high potential energy and very short α-carbon distance. Moreover, allosteric -RHStaple disulfides often exist in the -LHStaple configuration in different NMR models. The rarity of -LHStaple disulfides in X-ray structures is consistent with the finding that disulfides in crystallized proteins generally have lower strain energy than those found in solution structures. We suggest that the -LHStaple is an allosteric configuration.

## Methods

All structures released in the protein databank [34] as of June 20, 2006 were analyzed. Disulfide bonds in structures were determined by the presence of an SSBOND line in the PDB file. NMR structures were analyzed once, using the first model listed as the representative structure. The files were then separated into each individual model and analyzed.

Determination of the dihedral strain energy (DSE) was performed as described previously [8]. Briefly, the DSE of each disulfide was predicted from the magnitude of the five χ angles that define the disulfide using the empirical formula [17,18]:

DSE (kJ.mol^-1^) = 8.37(1+cos3χ_1_) + 8.37(1+cos3χ_1_') + 4.18(1+cos3χ_2_) + 4.18(1+cos3χ_2_') + 14.64(1+cos2χ_3_) + 2.51(1+cos3χ_3_)

χ_1 _is the dihedral angle about the C_α_-C_β _bond, χ_2 _about the C_β_-S_γ _bond, χ_3 _about the S_γ_-S_γ_' bond, χ_2_' about the S_γ_'-C_β_' bond and χ_1_' about the C_β_'-C_α_' bond. This relationship has been shown experimentally to reflect the amount of strain in a disulfide bond [19-22].

## Authors' contributions

Both authors made substantive contributions to conception and design of the study, and analysis and interpretation of data.
